# A Spatial-Temporal Multi-Feature Network (STMF-Net) for Skeleton-Based Construction Worker Action Recognition

**DOI:** 10.3390/s24237455

**Published:** 2024-11-22

**Authors:** Yuanyuan Tian, Sen Lin, Hejun Xu, Guangchong Chen

**Affiliations:** 1 School of Civil Engineering and Architecture, Wuyi University, Jiangmen 529020, China; yytian@wyu.edu.cn; 2 School of Business, East China University of Science and Technology, Shanghai 200231, China; linsenjn@ecust.edu.cn; 3 School of Civil Engineering and Architecture, Jiangsu University of Science and Technology, Zhenjiang 212100, China; 202300000210@just.edu.cn; 4 School of Management, Shanghai University, Shanghai 200444, China

**Keywords:** construction worker, action recognition, 3D skeleton, deep learning algorithm

## Abstract

Globally, monitoring productivity, occupational health, and safety of construction workers has long been a significant concern. To address this issue, there is an urgent need for efficient methods to continuously monitor construction sites and recognize workers’ actions in a timely manner. Recently, advances in electronic technology and pose estimation algorithms have made it easier to obtain skeleton and joint trajectories of human bodies. Deep learning algorithms have emerged as robust and automated tools for extracting and processing 3D skeleton information on construction sites, proving effective for workforce action assessment. However, most previous studies on action recognition have primarily focused on single-stream data, which limited the network’s ability to capture more comprehensive worker action features. Therefore, this research proposes a Spatial-Temporal Multi-Feature Network (STMF-Net) designed to utilize six 3D skeleton-based features to monitor and capture the movements of construction workers, thereby recognizing their actions. The experimental results demonstrate an accuracy of 79.36%. The significance of this work lies in its potential to enhance management models within the construction industry, ultimately improving workers’ health and work efficiency.

## 1. Introduction

The construction industry is crucial to a country’s prosperity, health, and quality of life [1,2], significantly contributing to GDP in industrialized nations—approximately 25–26% of China’s GDP in 2017, 9.3% in Hong Kong, and 5% in South Korea in 2012 [3,4]. It plays a vital role in economic and societal development, creating millions of jobs globally, and is one of the largest industrial sectors, employing about 7% of the world’s working population and projected to generate around 864,700 new jobs by 2026 [5]. However, it is also one of the most dangerous industries, with construction workers facing numerous hazards such as work-related musculoskeletal disorders (WMSDs) and a high rate of fatalities and injuries [6]. The industry accounts for 16.4% of all occupational injury fatalities worldwide [7] and has a 71% higher rate of non-fatal injuries than other industries [8]. In China [9], 2019 saw 734 construction accidents resulting in 904 deaths, a significant increase from the previous year. High accident rates, often linked to worker-centered issues, highlight the urgent need for effective safety management [10].

With the rapid development of information technologies in recent years, construction site management approaches have been further improved, especially in worker behavior recognition. Worker recognition is considered middle-level according to the complexity level of human behavior [11]. The early studies focused on machine learning with feature extraction procedures [12], such as RGB video-based and image-based. For instance, Gong et al. [13] used the Bag-of-Video-Feature-Words model integrated with Bayesian learning methods to analyze video data and classify construction workers. Abhinav Peddi et al. [14] used images in real-time to generate human poses associated with worker productivity measurement systems on a bridge construction project. Significantly, the working status of a worker is divided into three categories: effective work, ineffective work, and contributory work. Convolutional networks are used to identify activities. Luo et al. [15] used convolutional networks to encode spatial and temporal video features, achieving 80.5% accuracy in identifying and marking worker behaviors, despite challenging video conditions.

Recently, with the development of motion capture systems, researchers are gradually focusing more on three-dimensional (3D) motion information, which is acquired through two processes: RGB-D camera resources and wearable sensors. Beyond still images, this type of data provides a wealth of information about human movements, such as skeleton data, angle information, and key joint trajectories between time intervals. Furthermore, deep learning algorithms have been used in this system for action recognition, which significantly improves the accuracy of the recognition. For example, Ray and Teizer [16] proposed automating human posture estimation and monitoring workers’ ergonomic motions using a Kinect range camera. Specifically, they used a predefined ergonomic rule based on the National Institute for Occupational Safety and Health (NIOSH) as the basis for categorizing tasks, which included standing, squatting, bending, and crawling. Han and Lee [17] proposed using stereo cameras abstracting 2D human skeleton information, which is reconstructed in a 3D coordinate to detect workers’ unsafe actions automatically.

Researchers and practitioners have been endeavoring to improve safety in the construction industry in recent years. Furthermore, the development of computer vision techniques and deep learning algorithms provides strong technical support for quickly identifying construction worker behavior and achieving safety and health monitoring of construction site workers. However, most research based on small-scale datasets considers workers to always be dynamic and the most difficult to control on construction sites, especially because the dynamic and complex environment hides a series of dangers to the workers’ occupational safety and health. Furthermore, traditional worker management is inadequate to fully face the challenges of modern construction sites. Therefore, this research aims to improve construction workers’ accuracy and reliability based on the specific integrated large skeleton dataset and promote practical application ability with the latest improved recognition deep algorithms.

In this study, we aimed to utilize six 3D skeleton-based features to capture the local features of individual joints and the crucial contour features of significant joints for effective monitoring and capturing of the movements of construction workers. In detail, the four types of skeleton topology graphs not only preserve the original physical connections of the human body but also enhance the comprehensive enrichment of input features. Moreover, we incorporate velocity and acceleration as novel temporal features, fused with spatial features, enhancing the depth of information. Through the multi-stream fusion strategy, the model captures broader connections and features. The main contributions of this study can be summarized as follows:

➢ The study introduces the Spatial-Temporal Multi-Feature Network (STMF-Net), which incorporates GCN and TCN models to learn spatial-temporal feature sequences. Through GCN, the network can aggressively extract the node features from their neighbors at the spatial level. Through stack-TCN, the network can continuously extract sequence features at the temporal level. Fusing them could help the model extract robust features and boost recognition ability.
➢ For spatial features, we designed four different hierarchical skeleton topologies (Body-level, Part2-level, Part5-level, Joint-level) and utilized a graph convolutional network to extract features. In particular, the innovative joint-level structure is proposed. This strategy selects the root joint as the center, connecting all other joints to it, forming a star-like topology. This topology graph significantly reduces distances between nodes and captures more detailed features.
➢ The study adopted a spatial-temporal two-step fusion strategy, replacing the naive six-stream direct fusion strategy, to ensure optimal fusion performance by balancing the independent learning of feature streams and adequately correlating the fusion stream.

## 2. Literature Review

In this section, we show the structure of the human skeleton. Next, we introduce the common skeleton representation approaches, including joint-based and part-based methods. Finally, recent research focuses on Deep Neural Networks (DNN), compared with the conventional Machine Learning (ML) methods, which are data-driven approaches.

### 2.1. Human Skeleton Model

#### 2.1.1. Human Skeleton Structure

The human skeleton consists of over two hundred skeletal nodes, each possessing a certain degree of freedom. Considering all these nodes would lead to an exceedingly complex model, making it a challenging task to use for human action recognition. Many researchers have proposed simplified versions of the human skeleton model to address this. For instance, Microsoft Research developed the Kinect sensor [18], which employs 20 key joints to represent the entire skeleton. From an outline perspective, this simplified model closely matches the human body’s contour. Moreover, the algorithm’s lower complexity allows a real-time estimation of joint positions from a single depth frame, significantly simplifying the skeleton model and ensuring minimal distortion while expressing an action.

**Figure 1:**
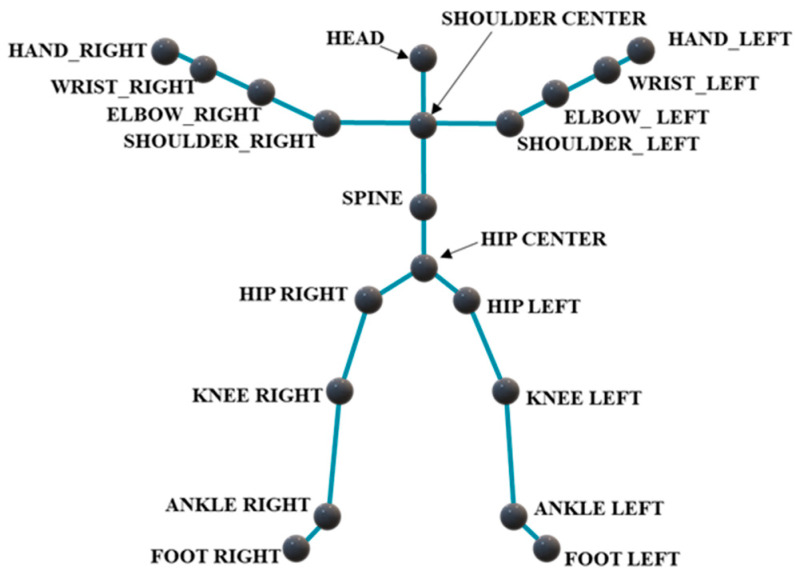
The 20 joint locations of the human body from Kinect.

As shown in Figure 1, this image represents the skeletal node map obtained from Kinect. It contains a total of 20 key joints. The image shows that the human skeleton can roughly reflect the body’s contour and the relationships between various joints.

#### 2.1.2. Spatial-Temporal Features of Skeletons

In recent years, with the development of recent electronic technology, researchers have gradually focused more on spatial-temporal information in various fields such as image-based tasks [19], gesture recognition [20], and human action recognition [21,22,23,24,25,26].

Initially, researchers utilized 3D skeleton models and motion sensing to develop automated human motion recognition models based on spatial-temporal features. Initially, methods primarily relied on Convolutional Neural Networks (CNNs) and Recurrent Neural Networks (RNNs) to extract spatiotemporal information from skeleton sequences for action recognition. CNN-based approaches emphasize the extraction of spatial information, representing temporal dynamics and skeleton joints as rows and columns in skeleton sequence representations. For instance, Li et al. [21] mapped skeleton images containing both temporal and spatial information by dividing the human body into five main parts in each frame and mapping these parts into 2D images as inputs. Li et al. [22] further introduced a new representation from geometric algebra called shape-motion representation, highlighting the importance of both joints and bones in the human body. However, using only CNN-based models to achieve robust recognition accuracy remains challenging. RNN-based methods, on the other hand, focus on encoding skeleton data that is rich in input features. For example, Liu et al. [23] proposed a novel tree-structure-based traversal input feature to rearrange the order of joints, thus ignoring the kinetic dependency relations between adjacent joints to recognize daily human activities. Similarly, Feng et al. [24] introduced two simple geometric feature vectors and normal vectors based on 15 joints as input. Wang et al. [25] incorporated three primitive geometries—joints, edges, and the normal vector of surfaces—as inputs to multiple layers of bidirectional LSTM deep networks to recognize and detect human actions across three different datasets.

Recently, GCN-based models have demonstrated a strong capability to capture the characteristics of 3D skeleton data, based on the natural topological graph of the human body. Yan et al. [26] introduced the ST-GCN network, leveraging GCNs to extract spatial features from skeleton data and TCNs to capture temporal dynamics for human action recognition. Their approach involved constructing a spatial topology graph with joints as vertices, connected based on natural human body structures, and using time as graph edges. Building on this concept and considering the unique motion features of construction workers (such as partial body movements and high hand correlation), we adopted multi-stream fusion GCN-TCN deep learning algorithms. Our model not only captures the inherent properties of 3D skeleton data but also integrates various spatial-temporal features, enhancing the automated identification of construction activities.

### 2.2. Features Based on Skeletons

Existing skeleton-based features for action recognition can be classified into two categories: joint-based features and part-based approaches.

#### 2.2.1. Joint-Based Approaches

Previously, skeleton-based features mainly focused on using joint coordinates as the basic units to represent skeleton data. The straightforward feature is the 3D joint coordinates [27,28], which can be concatenated. Additionally, some studies used the relative joint displacements as input features, such as the pairwise relative position study [29], joint orientation study [30], joint displacement relative to a reference center joint study [31,32], and the study using a collection of various features [33] to conduct representing 3D human skeletons for action recognition. Furthermore, some researchers used more comprehensive geometric features to improve the recognition of actions, such as the geometric relationship between the joint and the plane constructed by skeleton joints [34] or the vectors and normal vectors based on the input joints [24].

#### 2.2.2. Part-Based Approaches

Another mainstream extracting skeleton feature is based on segmenting the human body into different parts, which mainly considers the natural connection between human body structures. For example, Wang et al. [35] and Du et al. [36] divided the human body into several parts, such as the head, left/right arm, and left/right leg, according to human anatomy, to effectively characterize human actions. Similarly, referencing [37,38], they constructed features to represent these segments of the human body. Specifically, they partitioned the entire body into different areas, including the upper left arm, upper axial, upper right arm, lower axial, lower left leg, and lower right leg. Additionally, Liu et al. [39] emphasized the significance of anatomical segmentation in accurately modeling and recognizing human actions across various applications. Their method involved dividing the human body into four salient subgraphs—left arm, right arm, left leg, and right leg—to enhance the granularity and accuracy of action recognition. They formed seven additional intra-part graphs with non-salient parts. These studies divide the whole human body into different parts, considering the physical structure of the human body to explore more obvious skeleton features.

### 2.3. Skeleton-Based Action Recognition Algorithm

#### 2.3.1. Deep Learning Algorithm

Worker action recognition is a meaningful and challenging task in construction. Recently, there have been many studies that have focused on using deep learning algorithms to automate the extraction of information from skeleton data. Recurrent Neural Networks (RNNs) are the most common algorithm for skeleton-based action recognition due to their superiority in dealing with sequential data. Convolutional Neural Networks (CNNs) normally process the skeleton data into image format. For RNN-based methods, skeleton sequences are treated as natural time series of joint coordinates, making RNNs suitable for processing due to their structure. Additionally, advanced RNN-based methods like Long Short-Term Memory (LSTM) and Gated Recurrent Unit (GRU) have improved temporal context learning for skeleton-based action recognition. CNNs complement RNNs by focusing on spatial cues within input data. Recently, graph convolutional neural networks (GCNs) have been shown to be effective for skeleton data, which naturally form a topological graph structure with joints as vertices and bones as edges. This survey mainly focuses on GCNs for their effective representation of graph-structured data.

The GNNs model was first introduced in [40] as a generalization of recursive neural networks to deal with general graphs. Recently, the GCN model has been frequently adopted in skeleton-based tasks due to the effective representation of the human body structure in non-Euclidean space. Normally, the graph used in GCN-based models is manually constructed, relying mainly on the physical intra-links of the human structure, which correspond to the fixed adjacency matrix used in the model [26,41]. In this setting, each joint has the same weight during message passing and only follows the pre-fixed edge connections, which may be insufficient to describe all connections due to the diversity of samples in action recognition tasks. To overcome this limitation, a basic component in transformers called the self-attention mechanism [42] was introduced for its well-known capability of reweighting. There are already some researchers who attempt to establish implicit connections among physically non-adjacent joints [43,44,45]. For instance, Shi et al. [45] introduced an attention-enhanced adaptive GCN that conceptualized the learning process using two trainable matrices tailored for action recognition tasks. Meanwhile, Chan et al. [43] suggested that explicit graph relations might not accurately represent actual dependencies, proposing an alternative method to establish implicit connections and appropriately balance the weights for each action. To capture the hidden spatial dependency in multiple joints of human movement, a simple yet effective method to learn novel connections in the spatial-temporal graph was proposed to explore the genuine relation.

#### 2.3.2. Multi-Stream Neural Network

Recent studies have increasingly focused on multi-stream neural networks to boost action recognition performance. Initially, many researchers explored two-stream neural network approaches. For example, Wang and Wang [46] introduced an innovative two-stream RNN architecture that simultaneously captures temporal dynamics and spatial configurations in skeleton data, demonstrating its effectiveness in action recognition. Following this, Jia et al. [47] proposed a two-stream TCN architecture with 12 blocks to tackle challenges in skeleton-based human action recognition. In a similar vein, Li et al. [48] created a two-stream fusion network by combining skeleton coordinates with their temporal differences, improving action recognition performance. Shi et al. [49] proposed a two-stream framework based on GCNs, which adaptively parameterizes both first-order and second-order information, including bone-related data, to enhance action recognition. This approach marked a shift towards multi-stream networks. Shi et al. further developed a multi-stream attention-enhanced adaptive graph convolutional neural network (MS-AAGCN) [45], incorporating bone length and orientation as second-order information, which significantly improved accuracy. Similarly, Li et al. [50] designed a six-stream fusion network that independently processes six data modalities, including joints, bones, their movements, and relative positions, to further enhance action recognition capabilities.

## 3. Methodology

**Figure 2:**
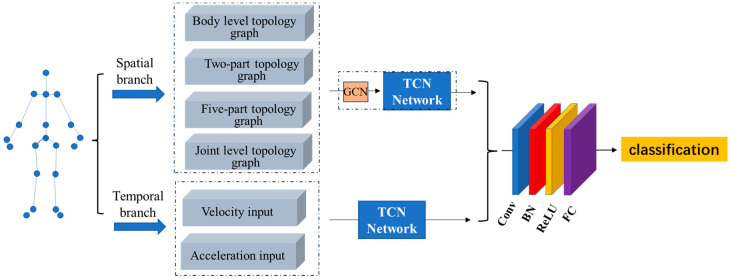
Illustrates the pipeline of the proposed overall model.

As illustrated in Figure 2, the proposed fusion network architecture consists of two primary branches: the spatial branch and the temporal branch. The joint-level branch processes input from four novel topology graphs (Body-level, Part2-level, Part5-level, and Joint-level). In this branch, the topology graphs are initially input into GCNs to effectively extract node features from neighboring nodes at the skeleton level. These features are then passed to a TCN to capture the sequence dynamics and enhance the model’s ability to recognize construction worker actions. For the temporal branch, the velocity and acceleration features are fed into the TCN-based model instead of using raw joint positions to capture temporal patterns more effectively. Finally, the fusion of both branches enables the model to extract robust features from both spatial and temporal domains, thereby improving its recognition performance.

### 3.1. Pipeline of the Proposed Sequence Network

**Figure 3:**
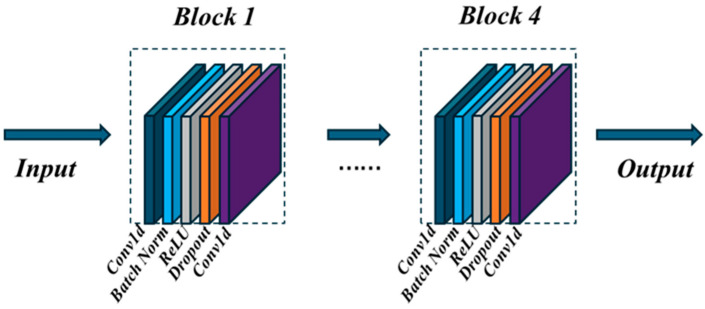
Overview of the proposed TCN architecture network.

Each stream is composed of four blocks which are connected by kernel size 1 temporal convolution layers. The output of blocks B1, B2, B3, and B4 are 32, 64, 128, and 256, respectively. All blocks are connected in series. The input data is normalized by a BN layer at the beginning of the network. The detailed architecture of the blocks is shown in Figure 3.

The temporal convolutional network is a network composed entirely of convolutional structures. It has achieved good results in sequence modeling tasks without using recurrent structure. The temporal convolutional network can be considered as a combination of one-dimensional convolution and causal convolution and is written as

(1) $$H^{\left(l+1\right)}=H^{\left(l\right)}W^{\left(l\right)}+{Bias}^{\left(l\right)}$$

where $H^{\left(l+1\right)}$ is the output feature of layer l, and $H^{\left(l\right)}\in R^{input\_size}$ represents the input size. The $W^{\left(l\right)}$ and $B^{\left(l\right)}$ denote the learnable parameter matrixes and the bias vectors, respectively.

In a GCN, the skeleton graph is represented by the adjacency matrix A. If skeletal joints $J_{i}$ and $J_{j}$ are connected, $A_{ij}$ is set to 1; otherwise, it is 0. Different adjacency matrices represent various skeleton topologies. Each skeleton frame is a graph G=(V,E), where V are joints (spatial features) and E are edges (structural features). Based on skeleton data X and adjacency matrix A, the GCN convolution operation is formulated in Equation (2).

(2) $$f_{out}=\sigma \;(D^{-\frac{1}{2}}\tilde{A}D^{-\frac{1}{2}}f_{in}W)$$

where $\tilde{A}=A+I$ is the adjacency matrix of graph G with self-connections identity matrix I. D is the degree matrix of $\tilde{A}$. W is the learned weight matrix and σ(·) denotes the activation function.

### 3.2. Input Feature

#### 3.2.1. Intra-Frame Input

For actions related to whole-body movement, the whole set of joints from the body structure can be used; however, for actions only related to partial-body movement, a subset can be selected. Typically, human actions can range from simple to complex, involving different parts of the body. Simple actions, such as punching forward or kicking, primarily rely on the movement of specific limbs—the arms for punching and the legs for kicking. Other actions, like bending down, involve the upper body. Complex actions, such as running and swimming, require a coordinated effort of the arms, legs, and trunk. To accurately recognize a wide variety of human actions, it is essential to model the movements of these individual parts and understand their interactions and combinations. This approach ensures a comprehensive understanding of both simple and intricate actions.

**Figure 4:**
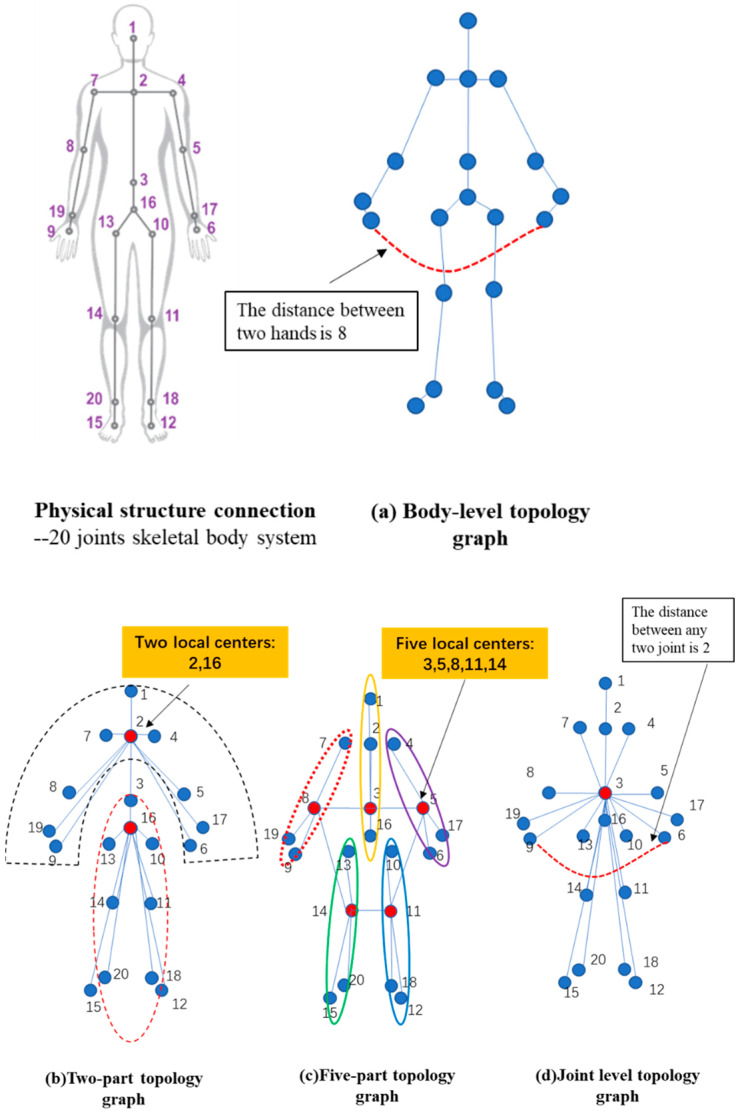
Representation of four skeletal typologies. The blue circles represent skeletal joints, and the red circles represent local centers (1 = Head, 2 = Neck, 3 = Spine, 4 = Left shoulder, 5 = Left elbow, 6 = Left hand, 7 = Right shoulder, 8 = Right elbow, 9 = Right hand, 10 = Left hip, 11 = Left knee, 12 = Left foot, 13 = Right hip, 14 = Right knee, 15 = Right foot, 16 = Hip center, 17 = Left wrist, 18 = Left ankle, 19 = Right wrist, 20 = Right ankle).

Currently, most human skeleton topologies use a natural human connection approach to connect skeletal joints. Based on the whole-part or the hierarchical level of the human body, we proposed four different topological graphs, as shown in Figure 4. The first hierarchy (1) is called the “Body-level topology graph”, which is the traditional skeleton graph structure and is based on the physical structure of the human skeleton. The second hierarchy (2) is called the “Two-part topology graph”, which selects two local centers in this graph by dividing the human body into two parts, including an upper region, and a lower region. The third hierarchy (3) is called the “Five-part topology graph”, which selects five local centers that correspond to the five most common parts of the human body. The fourth hierarchy (4) is called the “Joint-level topology graph”, which involves the root joint being taken as the center, and all other joints are connected to this joint. This topology graph references the classical star structure, which could greatly reduce the distances between any two nodes and characterize more detailed features, so is called the Joint-level topology graph. For example, in the Body-level topology graph, the distance between two hands is 9, while the distance between the right hand joints and the left hand is 2. Overall, the main idea underlying these proposed skeletal typologies is to divide the parts of the body according to a hierarchical structure.

#### 3.2.2. Inter-Frame Input

**Figure 5:**
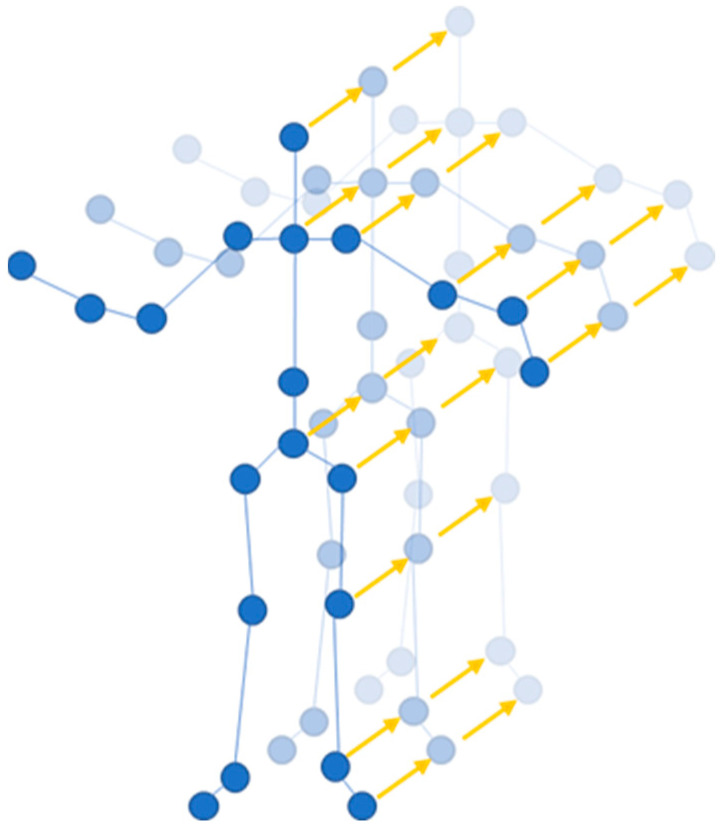
Temporal displacements (The blue circles represent skeletal joints, and the yellow arrows represent offsets of joints).

To capture robust motion features, this study utilized the offsets of joints over two temporal scales to better comprehend the movements of the skeleton, as shown in Figure 5. Specifically, the raw skeleton at frame t is represented as $P^{t}\in R^{N\times 3}$, where N is the number of joints, $P_{i}^{t}\in R^{3}$ denotes the 3D coordinate of i−th joint at time t. Then the joint accelerations at time t can be calculated as follows: $A^{t}=V^{t+1}-V^{t}$, where $V^{t}=P^{t+1}-P^{t}$. $V^{t}$ and $A^{t}$ can be considered as the first-order and second-order derivative of the joint coordinates, respectively.

## 4. Experiment

### 4.1. Dataset and Implementation Details

The CML dataset [51] used in this research contains more than 73 types of actions in four fundamental categories of activities, including 12 production activities, 38 unsafe activities, 10 awkward activities, and 13 common activities. The dataset of 61,275 samples was split as follows: 70% training, and 30% testing, with training data further split into 60% training and 10% validation. The model was trained on a desktop computer with an i7-11700 CPU at 2.50 GHz and a GeForce GTX 3060Ti GPU. The equipment was sourced from Lenovo, located in Beijing, China. We used the Adam [52] optimizer to automatically adjust the learning rate during training, with a weight decay set to 0.0005. The batch size was 256, adjusted based on GPU memory capacity. An initial learning rate of 0.00001 was used in preliminary experiments. To mitigate overfitting, we incorporated Batch Normalization [53] and a dropout layer with a probability of 0.5. The loss function is cross-entropy, and the weight decay was set to 0.0001.

### 4.2. Overall Performance of Multi-Stream Network

#### 4.2.1. The Overall Performance of Single Stream

**Figure 6:**
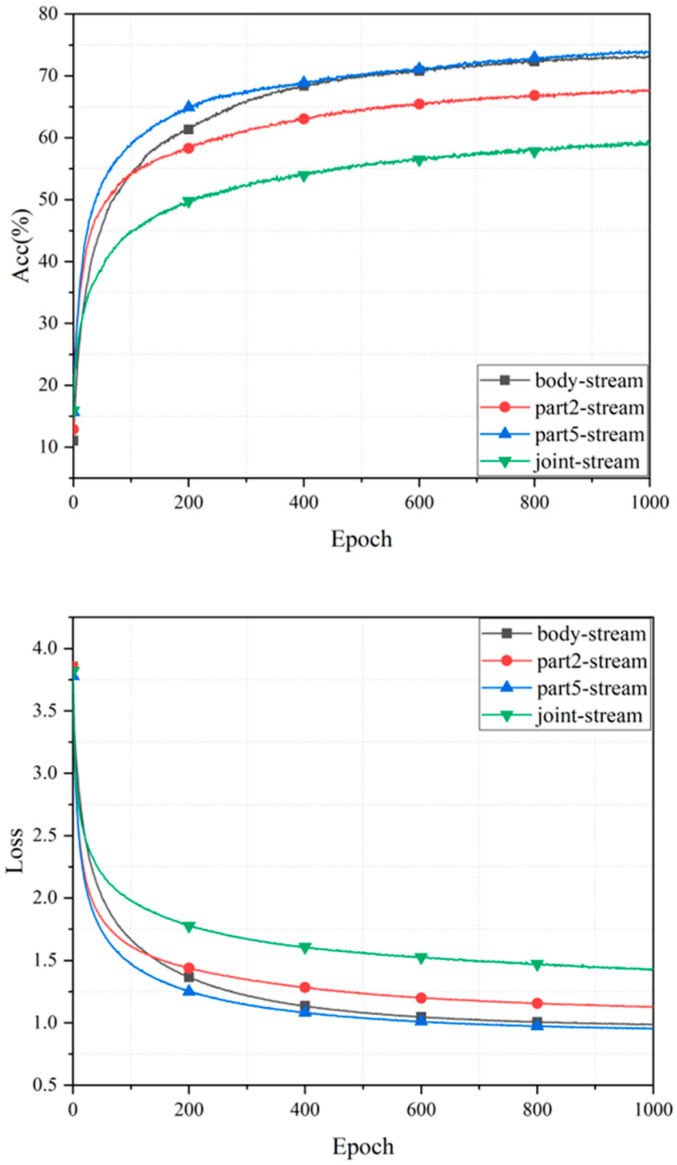
The validation set of the four single streams, showing accuracy and loss over the training epochs.

In this section, we evaluate each single stream to investigate the impact of different topology structures. Figure 6 plots the convergence rate curves of four different topology graphs on the validation set during training. The curves indicate that the convergence rates of all four topology graphs converge relatively quickly, within 200 epochs, without any signs of overfitting. Figure 6 also summarizes the performance of the four input streams based on the CML dataset. The figure shows that the Part5-level input stream outperforms the others, achieving an accuracy of 65.12%, which is comparable to the Part2-level input stream at 500 epochs. Additionally, the Part2-level input stream, Joint-level input stream, and Part5-level input stream reach their maximum values faster than the Joint-level input stream. Ultimately, the Part5-level input stream achieves the highest accuracy, indicating that its topology graph is more informative than the others. More detailed results are shown in Table 1.

#### 4.2.2. The Overall Performance of Fusion Spatial Stream

**Figure 7:**
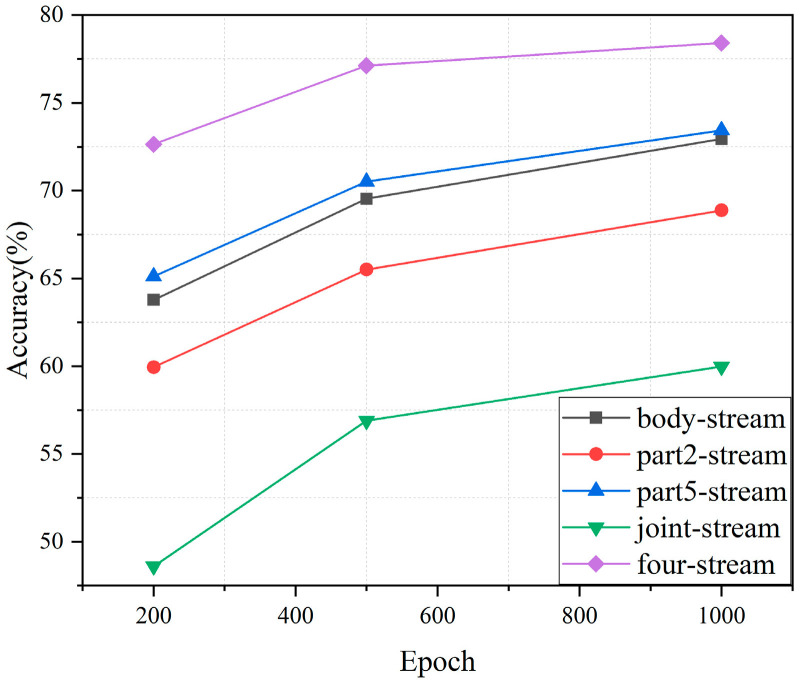
The accuracy of fusion spatial stream performance.

Figure 7 illustrates the accuracy of the fusion spatial stream performance. It is evident that the performance of the fused four streams significantly surpasses that of any single stream. Moreover, the fused four-spatial stream converges to its maximum value faster than the individual streams. At 200 epochs, its accuracy is approximately 7.51% higher than that of the Part5-level stream. This result indicates that fusing different levels of topology graphs effectively captures both global and local features, thereby enhancing the model’s performance in recognizing construction workers’ actions. This demonstrates the effectiveness of the hierarchical skeleton topology fusion strategy.

#### 4.2.3. The Performance of the Spatial-Temporal Fusion Data Stream

**Figure 8:**
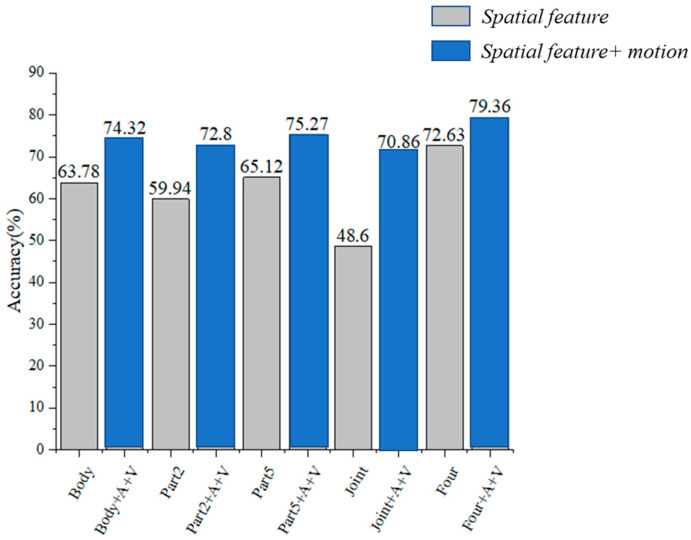
Validation set’s accuracy and loss over training epochs. (“A” and “V” represent the first-order and second-order derivative of the joint coordinates, respectively).

Figure 8 compares the performance of different spatial streams with and without the motion stream. The results indicate that the combination of four spatial features with the motion stream outperforms other feature sets. Additionally, compared to previous experiments, it is evident that motion data significantly enhances recognition accuracy. Experiments conducted with each single stream input, both with and without the motion data stream, show accuracy improvements of approximately 10.54%, 12.86%, 10.15%, and 22.26%, respectively. Furthermore, the four-stream input alone achieved an accuracy of 72.63%. When combined with the motion data, the accuracy improved by 6.73%, ultimately reaching 79.36%. This underscores the critical role of motion data in improving recognition performance.

#### 4.2.4. Comparison with Other State-of-the-Art Methods

To assess the performance of our proposed model, we conducted a series of comparative experiments with established action recognition methods. Among these, the ST-GCN model [26] is particularly noteworthy, as it was the pioneering approach to utilize graphs for extracting dynamic information from human body skeletons, achieving high accuracy on benchmark datasets for skeleton-based deep learning. When we reproduced ST-GCN on the same dataset, our model showed comparable performance. Despite its accuracy, the complexity and training speed of the ST-GCN model cannot be overlooked. ST-GCN employs 9 GCN blocks for spatial and temporal graph convolutions, resulting in 16.2 G floating point operations (FLOPs). In contrast, our model requires only approximately 0.5 G FLOPs, demonstrating a significantly lighter framework for recognizing construction workers’ motions. To ensure a comprehensive evaluation, we also compared our method with three dynamic modeling approaches, including traditional RNN [54] and 1/2-layer LSTM [55], as presented in Table 2. Traditional RNN achieved the lowest accuracy at 72.71%, while both 1-layer and 2-layer LSTMs performed better but still fell short by 2.91% and 1.99%, respectively, compared to our model. This disparity may be attributed to the fact that these models primarily focus on the temporal dynamics of actions, without fully leveraging the spatial characteristics that are crucial for accurately recognizing the unique motions of construction workers.

## 5. Conclusions

This research proposes the STMF-Net model, which innovatively utilizes six 3D skeleton-based features, including a fourth hierarchy and the velocity and acceleration derivatives of joint coordinates, to monitor and capture the movements of construction workers, thereby recognizing their actions. Compared to complex deep learning networks, we replace traditional sequence recognition methods (e.g., LSTM and other RNN-based networks) with the lightweight Stack-TCN as the primary sequence network, enhancing efficiency and practicality in action recognition for construction environments. Overall, we adopt GCN and sequential TCN connections in this work, aiming for a more lightweight network for worker action recognition.

Specifically, we presented a multi-feature network (MF-Net), a robust and efficient system for learning six individual spatial-temporal feature sequences (Body-level stream, Part2-level stream, Part5-level stream, Joint-level stream, Velocity stream, and Acceleration stream) derived from raw skeleton data. Additionally, we incorporated spatial-temporal features to correlate high-level feature maps from multiple streams, marking it as the first network to combine more than three geometric descriptors. We balanced independent learning of feature streams with adequate correlation in the fusion stream to ensure optimal fusion performance. Our experiments demonstrated the effectiveness of incorporating additional input features, resulting in notable improvements in recognition accuracy. Among the inputs, the five-part topology graph outperformed others, validating the design’s rationale. Furthermore, all spatial features showed superior results when combined with the motion data stream. Ultimately, the four-stream input, in conjunction with the motion data, achieved an accuracy of 79.36%.

As action recognition techniques advance in construction, developing efficient and lightweight action recognition systems for workers is crucial. Deep learning frameworks for skeleton-based recognition are key to ensuring worker safety and productivity. This research highlights the STMF-Net strategy and the fusion of six spatial-temporal features, significantly enhancing action recognition and contributing to improved workplace safety and efficiency in the construction industry.

## Figures and Tables

**Table 1:** Comparison of action recognition performance of different skeletal topology presentation streams.

Epoch	Body-Stream Acc (%)	Part2-Stream Acc (%)	Part5-Stream Acc (%)	Joint-Stream Acc (%)
200	63.78	59.94	65.12	48.6
500	69.53	65.51	70.51	56.89
1000	72.94	68.87	73.43	59.98

**Table 2:** Comparison of the performance with existing methods.

Algorithms	Acc (%)
ST-GCN	79.51
2-layer LSTM	77.37
1-layer LSTM	76.45
Traditional RNN	72.71
Our approach	79.36

## Data Availability

All data generated or analyzed during this study were included in this published article.
